# Correction to: Supplying Relief: Civil Medical Assistance during the Korean War, 1950–3

**DOI:** 10.1093/shm/hkaf068

**Published:** 2025-08-19

This is a correction to: Dongkue Lee, Mark Harrison, Supplying Relief: Civil Medical Assistance during the Korean War, 1950–3, *Social History of Medicine*, 2025, hkaf028, https://doi.org/10.1093/shm/hkaf028

Acknowledgement of funding support from the National Research Foundation of Korea was omitted from the version originally published, and has been added.

